# Food Industry By-Products as Natural Preservatives: Supporting Adolescent Food Literacy and Critical Food Choices

**DOI:** 10.3390/nu18121859

**Published:** 2026-06-09

**Authors:** Paula Silva

**Affiliations:** 1 Department of Microscopy, School of Medicine and Biomedical Sciences (ICBAS), University of Porto (U. Porto), Rua Jorge Viterbo Ferreira 228, 4050-313 Porto, Portugal; psilva@icbas.up.pt; 2 iNOVA Media Lab, ICNOVA-NOVA Institute of Communication, NOVA School of Social Sciences and Humanities, Universidade NOVA de Lisboa, 1069-061 Lisbon, Portugal

**Keywords:** food industry by-products, natural preservatives, food waste valorization, adolescent food literacy, food labels, sustainable food choices, circular food systems

## Abstract

This review aims to critically examine food industry by-products as potential sources of natural preservatives and to discuss how this evidence can be translated into adolescent food literacy, label interpretation, and critical food choices. Adolescents are increasingly exposed to food labels and claims about “natural,” “clean-label,” “upcycled,” “sustainable,” and “circular” foods, which may not always be transparent or supported by sufficient evidence regarding their safety, efficacy, sensory quality, consumer acceptance, or environmental benefit. Therefore, they need more than nutritional information; they need to interpret labels, question sustainability claims, and understand how food innovations are produced, tested, communicated, and regulated. Food by-products such as fruit and vegetable pomaces, peels, seeds, skins, olive and wine residues, cereal by-products, coffee silverskin, and cocoa residues are promising resources for clean-label preservation and circular food systems because they may contain phenolics, flavonoids, carotenoids, anthocyanins, essential oils, pectin, dietary fibers, and other compounds with antioxidant, antimicrobial, coloring, stabilizing, and texturizing properties. However, the bioactive potential alone does not guarantee that a by-product-derived ingredient is safe, effective, acceptable, scalable, or sustainable. Its use requires extraction, stabilization, real-food validation, safety assessment, sensory optimization, regulatory compliance, and sustainability evaluation. The review concludes that by-product-derived natural preservatives are both technological resources and educational tools. Future research and education should connect food preservation, label interpretation, food safety, sensory quality, sustainability evidence, and consumer decision-making to empower adolescents as critical consumers and informed agents of change in sustainable food systems.

## 1. Introduction

Food waste is increasingly recognized not only as an environmental and economic problem but also as a missed opportunity for food system innovation. Large quantities of residues are generated along the agri-food chain during harvesting, processing, retail, and consumption. In fruit and vegetable processing, these residues include peels, seeds, skins, pulp, stems, leaves, pomace, and other fractions that are often discarded or used for low-value purposes, such as animal feed, composting, or energy production. However, these materials frequently retain high concentrations of bioactive compounds, including phenolic compounds, flavonoids, carotenoids, anthocyanins, vitamins, dietary fibers, essential oils, and other phytochemicals with antioxidant and antimicrobial properties. Rather than being viewed only as waste, food industry by-products can be reframed as valuable resources for developing functional ingredients, natural additives, and preservation strategies that are aligned with more sustainable food systems [1,2,3,4,5,6,7].

This shift is particularly relevant in the context of the growing consumer demand for foods perceived as natural, minimally processed, and free from synthetic additives. The clean-label movement has encouraged the food industry to search for recognizable, natural, and multifunctional ingredients that can maintain quality and safety while responding to consumer expectations. Bioactive compounds recovered from agro-industrial by-products are attractive in this context because they may act as natural antioxidants, antimicrobials, colorants, stabilizers, and functional ingredients. Their use can support shelf-life extension, reduce reliance on synthetic preservatives, and contribute to food products that combine technological performance and sustainability [8,9,10,11,12,13].

Among the most studied sources of preservative compounds are fruit and vegetable processing by-products, particularly pomace, peels, seeds, and skins. Citrus residues, grape pomace, pomegranate peel, apple pomace, tomato pomace, carrot pomace, banana peel, mango peel, avocado peel, onion skin, and berry pomace have been investigated as sources of phenolic compounds, carotenoids, pigments, dietary fibers, and other bioactive compounds. These compounds may contribute to food preservation by delaying lipid oxidation, inhibiting microbial growth, reducing quality deterioration, improving color stability, and enhancing the nutritional and functional profiles of food products [1,2,3,5,7,14].

The transition from a by-product to a preservative ingredient is technologically complex. This process usually requires raw material selection, stabilization, drying, extraction, purification or concentration, formulation, and incorporation into a food matrix or packaging system. Conventional extraction methods remain widely used; however, greener and more efficient technologies, including ultrasound-assisted extraction, microwave-assisted extraction, supercritical fluid extraction, enzyme-assisted extraction, and pulsed electric field processing, have gained increasing attention because they can improve extraction efficiency, reduce solvent use and processing time, and better preserve heat-sensitive compounds [15,16,17,18,19,20]. Once recovered, bioactive compounds must remain stable and active in real food systems. Therefore, encapsulation, spray drying, freeze-drying, edible coatings, and active packaging have been explored as stabilization and delivery strategies to help maintain food safety, quality, and shelf life [12,20,21,22,23,24].

Despite promising laboratory and pilot-scale evidence, translation into industrial and market applications remains limited. By-product-derived preservatives have been tested in meat, dairy, and bakery products, beverages, fresh produce, and active packaging systems. However, their performance depends on food matrix interactions, dose, stability, safety, sensory quality, consumer acceptance, cost, and regulatory requirements. Studies on meat, dairy, bakery products, and active packaging illustrate this matrix-dependent performance, while techno-economic and consumer studies show that cost, scale-up, labeling, trust, and acceptance remain decisive for commercial translation [12,25,26,27,28,29,30,31,32,33,34].

The sustainability rationale for this field is robust but must be critically demonstrated. Valorizing food industry by-products can reduce waste disposal burdens, create value-added ingredients, support circular food systems, and contribute to resource efficiency in the food industry. However, circularity should not be assumed solely because a by-product is used; extraction inputs, energy demand, solvent use, water consumption, packaging, transport, scale-up, and the fate of residual biomass should also be considered. Life-cycle, techno-economic, and eco-efficiency studies show that the environmental and economic value of by-product valorization depends strongly on the process design, scale, product yield, and integration of residual biomass streams [35,36,37,38]. In dairy products, fruit and vegetable pomace incorporation has also been linked to nutritional improvement, functional enrichment, waste reduction, and circular economy objectives, with some reviews explicitly connecting pomace valorization with SDGs 2, 11, and 12 [35,39,40,41,42].

However, the relevance of this topic extends beyond food technology and industrial innovation. It also has strong educational potential. Adolescents are current and future consumers, family influencers, peer communicators, digital citizens, and emerging citizens who will shape food choices, sustainability practices, and expectations of the food industry. Research on adolescent food literacy and food systems education shows that young people can develop competencies that go beyond nutrition knowledge, including the ability to understand food systems, reduce food waste, and engage with healthier and more sustainable diets [43,44,45,46,47,48,49].

For adolescents, the key educational message is not that all food waste can or should be eaten or that natural preservatives are automatically safe. Rather, the message is that science can help transform selected food industry by-products into safe, tested, and useful ingredients that may extend shelf life and reduce waste. This distinction is essential for responsible food literacy. It can help adolescents question labels, sustainability claims, and assumptions about naturalness, while supporting evidence-based communication within families, schools, and peer networks [43,44,49,50,51].

The aim of this narrative review is to critically examine food industry by-products as potential sources of natural preservatives and to explore how this evidence can be translated into adolescent food literacy. Specifically, the review addresses: (i) the main food industry by-products investigated as sources of preservative compounds; (ii) the bioactive compounds, extraction and stabilization technologies, food matrices, and active packaging applications reported in the literature; (iii) the safety, sensory, regulatory, consumer acceptance, economic, and sustainability challenges that limit translation into food applications; and (iv) the educational implications of this evidence for helping adolescents interpret food labels, question natural, clean-label, upcycled, and sustainability claims, and make more critical food choices.

This paper is a narrative review designed to integrate evidence from food science, food technology, sustainability, consumer research, and adolescent food literacy. The literature was identified through searches in PubMed, Scopus, Web of Science, Google Scholar, and relevant journal databases, complemented by backward and forward citation tracking of key publications. Priority was given to peer-reviewed articles, recent reviews, and studies addressing food industry by-products, bioactive compounds, natural preservation, extraction and stabilization technologies, active packaging, food applications, sensory and consumer acceptance, sustainability assessment, and adolescent food literacy. The review did not follow a systematic review protocol and did not aim to provide an exhaustive quantitative synthesis. Instead, it provides a critical and interdisciplinary synthesis of evidence relevant to understanding how by-product-derived preservation can support adolescent food literacy and critical food choices. The distinctive contribution of this review is its interdisciplinary framing: by-product-derived preservation is not presented only as a food technology strategy but also as a context for developing critical food literacy among adolescents who are increasingly exposed to naturalness, clean-label, upcycling, and sustainability claims.

## 2. Conceptual Framework: From Food Waste Valorization to Clean-Label Preservation and Adolescent Food Literacy

In this review, by-product valorization is conceptualized as a three-step process. The first layer is technological and concerns the recovery, stabilization, and application of bioactive compounds from food industry by-products. The second layer is systemic and concerns the contribution of these processes to waste reduction, resource efficiency, clean-label innovation, and circular food systems. The third layer is translational and concerns how scientific knowledge can be communicated to adolescents as current and future consumers, household influencers, peer communicators, and emerging citizens. The distinctive perspective of this review is that adolescents are not approached only as recipients of information but as potential agents of change who can question food labels, interpret sustainability claims, reduce avoidable waste, and support food innovations that are safe, transparent, and environmentally responsible.

**Figure 1:**
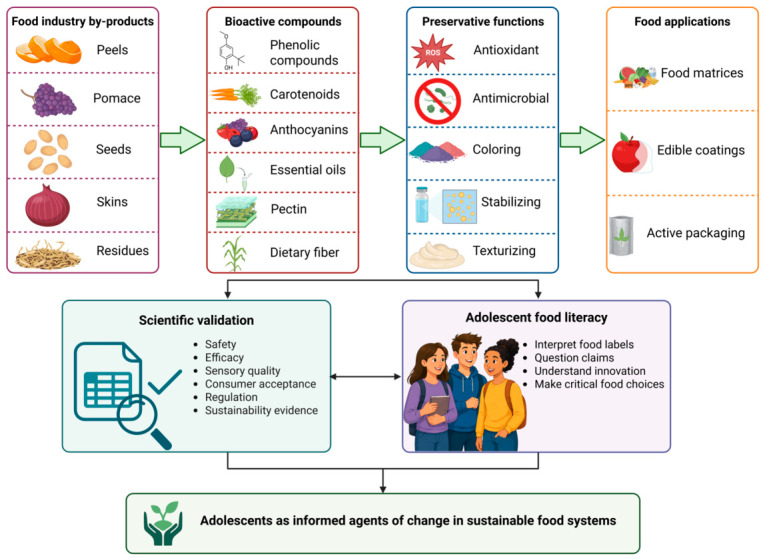
Conceptual framework linking food industry by-products, bioactive compounds, preservative functions, food applications, scientific validation, and adolescent food literacy. Created in BioRender. Silva, P. (2026) https://BioRender.com/x3x3unh.

The proposed framework follows a pathway from residue to functionality and from functionality to agency (Figure 1). At the technological level, food industry by-products such as peels, seeds, skins, pomace, stems, leaves, and processing residues may contain bioactive compounds with antioxidant, antimicrobial, anti-browning, coloring, stabilizing, and functional properties. These compounds can be extracted, stabilized, formulated, and incorporated into food matrices or packaging systems to extend shelf life, preserve quality, and reduce the use of synthetic additives. At the system level, this pathway contributes to circularity by transforming residual biomass into value-added ingredients and potentially reducing food loss through preservation. At the translational level, the same pathway can be converted into food literacy messages that help adolescents understand the scientific, environmental, economic, sensory, regulatory, and ethical dimensions of food waste valorization (Table 1) [43,44,49,52,53,54,55].

This framework moves the review beyond a purely technical description of by-product-derived preservatives. It emphasizes that the transition from food waste to clean-label preservation depends on sequential scientific and technological decisions: identifying and characterizing by-products, recovering and stabilizing bioactive compounds, testing them in real food matrices or packaging systems, and evaluating their effects on shelf life, food quality, safety, sensory performance, consumer acceptance, and sustainability [42,52,54,55,56,57]. At the same time, the framework highlights the translational value of this topic for adolescent food literacy by connecting food waste, preservation, clean-label innovation, food safety, sensory quality, regulation, and sustainability with the development of critical food-related questions [43,44,48,49,50,58].

## 3. Food Industry By-Products Investigated as Natural Preservative Sources

Food industry by-products investigated as sources of natural preservatives are highly diverse; however, the evidence is dominated by plant-derived residues, particularly fruit and vegetable processing by-products. These include pomace, peels, skins, seeds, pulp residues, stems, leaves, bran, shells, and residues generated after the extraction of juice, wine, oil, starch, or sugar. Fruit and vegetable by-products are especially relevant because they often retain phenolic compounds, flavonoids, carotenoids, anthocyanins, vitamins, minerals, dietary fibers, pectin, essential oils, and other phytochemicals with antioxidant, antimicrobial, coloring, stabilizing, and functional properties. Fruit pomace, in particular, has been described as a major by-product stream generated from apple, grape, citrus, and mango processing and contains dietary fiber, carbohydrates, phenolic compounds, polysaccharides, phytochemicals, natural antioxidants, and other health-promoting nutrients [1,2,5].

From the perspective of clean-label preservation, these by-products are attractive because they may provide multifunctional ingredients for food products. A single residue may contribute antioxidant compounds, antimicrobial fractions, natural pigments, texturizing fibers, stabilizing polysaccharides, or sensory-active components. This multifunctionality is valuable for food formulation but requires careful evaluation. A by-product cannot be considered a preservative source simply because it contains bioactive compounds. Its applicability depends on the composition, safety, extraction efficiency, stability, sensory impact, regulatory status, cost, and performance in a specific food matrix. This distinction is especially important for adolescent food literacy: the educational message should not be that “waste can become food” but that selected by-products can become useful ingredients only after scientific characterization, processing and validation [11,32,59].

### 3.1. Fruit By-Products

Fruit by-products are among the most frequently investigated sources of natural preservatives and functional compounds. The main residues include citrus peels and pomace, grape pomace, pomegranate peels, apple pomace, banana peels, mango peels, avocado peels and seeds, tomato pomace, and berry pomaces. In industrial fruit processing, pomace commonly consists of peels, pulp, flesh, skin, stems, and seeds. Its high moisture content makes it vulnerable to microbial spoilage, which explains why drying, milling, conditioning, extraction, or stabilization is often necessary before its use in food applications [1,2,5].

Citrus by-products are among the most extensively studied fruit residues. Citrus processing generates peels, pulp, membranes, seeds, juice vesicles, and pomace, which are rich in flavonoids, polyphenols, carotenoids, essential oils, pectin, dietary fiber, sugars, ascorbic acid, and trace elements [60,61,62]. Citrus peels and essential oils provide antioxidant, antimicrobial, flavoring, and packaging-related properties, whereas citrus pectin is widely used as a stabilizer, thickener, emulsifier, and gelling agent in food systems [60,63,64]. Recent applications include citrus pomace-enriched yogurt, where low levels of incorporation improved selected physicochemical, sensory, and microbiological properties, and pectin-based active films containing lemon essential oil for antioxidant and biodegradable packaging applications [39,65,66]. These examples show adolescents that preservation may occur either through ingredients added to food or through packaging systems designed to reduce spoilage.

Grape pomace is another major fruit by-product, especially in wine and juice production. It generally contains pressed skins, seeds, residual pulp, and stems and is rich in dietary fiber and phenolic compounds, including hydroxycinnamic acids, anthocyanins, flavonol glycosides, flavanols, condensed tannins, and proanthocyanidins. Grape skins are particularly rich in anthocyanins and flavonols, whereas seeds contain flavanols and oil. Its composition varies according to the grape variety, processing technology, seed-to-skin proportion, and extraction conditions, making standardization a central challenge [5,67]. Grape-derived residues have been tested in matrices where oxidation and microbial spoilage are relevant, particularly in meat and bakery products. Incorporating 0.5–1.0% grape pomace powder into beef model systems can enhance oxidative and microbial stability during refrigerated storage, while grape pomace-enriched biscuits illustrate the potential of this residue in bakery applications [34,67,68,69]. However, these examples also show that concentration, matrix, storage time, and sensory consequences determine whether a by-product can function as a clean-label preservative.

Pomegranate peels have emerged as highly relevant natural preservatives. They are rich in phenolics, flavonoids, tannins, ellagitannins, punicalagin, and ellagic acid, and have been investigated for their antioxidant and antimicrobial functions. Recent studies have tested the use of pomegranate peel powder or extracts in bakery, meat, and fish products. In muffins, 8% pomegranate peel powder exhibited antifungal activity against *Penicillium* spp. and *Aspergillus* spp., increased fiber, total phenols, tannins, and antioxidant activity, and produced textural changes, although the taste became slightly bitter. Pomegranate peel and olive leaf extracts have been investigated as natural additives to limit microbial growth and oxidative deterioration in fresh meat. In thornback ray sausages, pomegranate peel extract exhibited stronger protective effects than ascorbic acid and was proposed as a natural additive for refrigerated fish products [13,70,71].

Apple pomace, generated during apple juice, cider, and beverage processing, contains pectin, dietary fiber, phenolic compounds, sugars, minerals, and other bioactive fractions; however, its high moisture content requires stabilization before food use [72,73]. It has been tested in dairy and bakery applications, including yogurt and Greek yogurt, where apple pomace ingredients increased the phenolic content and antioxidant activity, although consumer testing showed that the most acceptable formulation was not necessarily the one with the highest pomace level [32,33,74]. Other fruit residues, including mango peels, banana peels, avocado peels and seeds, and berry pomaces, provide fiber, pectin, carotenoids, lipids, phenolics, and anthocyanins with potential functional value [75,76,77,78,79,80,81,82]. However, these compounds may be unstable or sensorially challenging, reinforcing the need to balance bioactive enrichment with stability, sensory quality, and consumer preferences.

### 3.2. Vegetable By-Products

Vegetable by-products include tomato peels and seeds, carrot pomace, beetroot peels, onion peels, broccoli leaves and stems, potato peels, cauliflower residues, and other trimming or peeling fractions. These residues may contain carotenoids, betalains, phenolic acids, flavonoids, glucosinolates, dietary fibers, pectin, vitamins, and minerals. Their preservative relevance is linked mainly to antioxidant activity, color protection, anti-browning effects, microbial inhibition, fiber enrichment, and food structure stabilization [83,84].

Tomato, carrot, beetroot, Brassica, potato, and onion by-products illustrate the functional diversity of vegetable residues. Tomato peels and pomace are relevant sources of lycopene and other carotenoids, whereas carrot pomace provides β-carotene and dietary fiber. Beetroot residues provide betalains and antioxidant pigments, and Brassica by-products may contain glucosinolates, isothiocyanates, phenolics, and antioxidant compounds [85,86,87,88,89,90,91,92]. These residues have been explored for use in beverages, yogurt, whey products, colorant applications, and antioxidant formulations; however, their use may affect pigment stability, taste, aroma, rheology, and visual acceptance. Potato and onion peels provide additional antioxidant and pigment compounds; however, safety and sensory issues, including glycoalkaloids, processing contaminants, strong color, odor, and flavor, must be considered [93,94,95]. These examples reinforce the central message that vegetable by-products become candidates for food use only after safety, composition, processing, and sensory evaluations.

### 3.3. Other Agro-Industrial Residues

Other agro-industrial residues investigated as sources of preservative or functional compounds include cereal bran, brewer’s spent grain, olive pomace, olive paste, olive mill wastewater, coffee silverskin, cocoa residues, seed cakes, nut skins, and pulse-processing residues. Although these materials are not always studied primarily as preservatives, they may contribute to antioxidant activity, fiber enrichment, microbial inhibition, texture modification, and active packaging functions [96,97]. Cereal bran and brewer’s spent grain are important sources of dietary fiber, β-glucans, phenolic acids, arabinoxylans, proteins, and antioxidants. Their use in bakery products is particularly relevant because bakery matrices can accommodate dry powders and fiber-rich fractions. However, the incorporation of these residues may affect dough rheology, texture, volume, color, bitterness, and consumer acceptance; therefore, their value as clean-label ingredients depends on formulation control and sensory optimization [98,99]. Olive oil by-products, including olive pomace, olive paste, olive leaves, and olive mill wastewater, are important sources of phenolic compounds, secoiridoids, hydroxytyrosol, tyrosol, oleuropein derivatives, and antioxidant activity. Their use has been explored in food preservation, including fresh meat, where olive leaf extract combined with pomegranate peel extract has been tested to control microbial growth and the oxidative deterioration of food. However, olive-derived residues also present formulation challenges, particularly bitterness, astringency, color changes, and sensory acceptability [67].

Coffee silverskin and cocoa residues broaden the range of by-products relevant to circular food systems. Coffee silverskin provides dietary fiber, chlorogenic acids, caffeine, and antioxidant compounds, whereas cocoa residues provide polyphenols, fibers, methylxanthines, and flavor-active fractions [97,100]. These materials are particularly useful for adolescent food literacy because coffee, chocolate, and bakery products are familiar. They allow discussion of how popular products generate hidden residue streams and how valorization may create both opportunities and risks.

### 3.4. By-Products for Active Packaging and Edible Coatings

A distinct group of applications involves by-product-derived extracts, essential oils, pectin, polysaccharides, and phenolic-rich fractions used in active packaging, edible coatings, and biodegradable films. Citrus residues are particularly relevant in this regard. Citrus essential oils have been incorporated into polysaccharide-based edible films and coatings, and lemon peel essential oils have been described as natural antibacterial and antioxidant agents. Citrus pectin, extracted from peels and other residues, is also relevant as a film-forming, stabilizing, and texturizing agent [65,66,101].

The active packaging route is important because it separates preservative action from direct ingredient addition. Active agents can be incorporated into packaging materials and released gradually to scavenge oxygen, inhibit microbial growth, reduce oxidation, or maintain quality. For adolescents, this is a valuable conceptual point: food preservation is not only about “what is inside the food” but also how the food is packaged, stored, transported, and protected. This helps connect by-product valorization with real food systems and the environmental question of whether a new packaging solution is genuinely more sustainable [12,54,102].

### 3.5. Critical Synthesis and Adolescent Food Literacy Relevance

The sources reviewed in this section show that fruit by-products, vegetable by-products, and other agro-industrial residues offer candidate compounds for clean-label preservation. Citrus peels, grape pomace, pomegranate peels, apple pomace, mango peels, banana peels, tomato pomace, beetroot peels, carrot residues, olive by-products, cereal bran, brewer’s spent grain, coffee silverskin, and cocoa residues illustrate that food system residues may retain biological and technological value. However, their readiness for food applications differs. Some, such as citrus pectin, are well-established ingredients. Other alternatives, such as citrus essential oils, pomegranate peel extracts, grape pomace powders, and anthocyanin-rich residues, are promising but require careful control of dose, stability, sensory impact, safety, labeling, and consumer acceptance.

For adolescents, this section provides a concrete entry point for critical food literacy. The classification of by-products by source helps them recognize where food residues come from: juice processing, wine production, fruit peeling, vegetable trimming, dairy formulation, bakery production, and packaging. Their preservative potential helps adolescents understand that science can transform selected waste streams into useful food preservation ingredients [44,49,51]. At the same time, these examples show that claims about naturalness, circularity, or upcycling require evidence from safety, efficacy, sensory, and sustainability assessments. Therefore, this review proposes that adolescents can be encouraged to ask which residue was used, which compound is responsible for the effect, whether it was tested in a real food system, which concentration is effective, whether it changes taste, color, or texture, whether safety was assessed, and whether the clean-label or sustainability claim is supported by evidence (Table 2).

This organization maintains the section aligned with the scope of the Special Issue by emphasizing valorization, bioactive compounds, food applications, active packaging, and circularity. Simultaneously, it reinforces the innovative angle of the review: by-products are not only technological resources but also educational objects through which adolescents can learn to connect food waste, preservation, food safety, clean-label innovation, sensory quality, and evidence-based sustainability.

## 4. From Bioactive Compounds to Food Applications: Functions, Technologies and Matrices

The preservative potential of food industry by-products depends on the bioactive compounds they contain, the technologies used to recover and stabilize them, and their performance in real food matrices. Phenolic compounds, flavonoids, tannins, anthocyanins, carotenoids, essential oils, pectin, dietary fibers, polysaccharides, and other phytochemicals are associated with antioxidant, antimicrobial, color-stabilizing, anti-browning, texturizing, and functional properties. However, these effects should not be interpreted as intrinsic guarantees of the efficacy of preservatives. A compound or extract that exhibits antioxidant or antimicrobial activity in vitro may behave differently when incorporated into meat, dairy, bakery products, beverages, fresh produce, or packaging materials. Its effectiveness depends on the dose, solubility, pH, water activity, lipid and protein interactions, oxygen exposure, storage conditions, processing, and sensory impact [52,54,55,56].

### 4.1. Preservative Functions of By-Product-Derived Compounds

Antioxidant activity is one of the most frequently reported functions of by-product-derived compounds. Phenolic compounds, flavonoids, tannins, anthocyanins, carotenoids, tocopherols, and related compounds may inhibit lipid oxidation, protein oxidation, pigment degradation, and quality deterioration. This function is particularly relevant in fat-containing foods, such as meat, fish, dairy products, nuts, and bakery products, where oxidation can reduce shelf life and negatively affect flavor, color, nutritional quality, and consumer acceptance [5,11,12,13,31,34].

Antimicrobial activity is another major preservation-related function of phenolic compounds. Essential oils, phenolic-rich extracts, tannins, pomegranate peel extracts, citrus-derived compounds, coffee silverskin extracts, olive mill wastewater polyphenols, and certain plant by-product powders have been investigated for their ability to inhibit spoilage microorganisms or foodborne pathogens. However, the antimicrobial efficacy of these agents is highly matrix-dependent. The same extract may perform differently depending on the food pH, water activity, fat content, protein content, storage temperature, packaging system, and initial microbial load. Therefore, antimicrobial activity should be evaluated in real food systems rather than inferred from inhibition zones or model assays [11,13,54,96,97].

Coloring, anti-browning, stabilization, and texturization functions are also relevant. Anthocyanin-rich residues, beetroot pigments, black carrot pomace, tomato lycopene, carrot β-carotene, and citrus-derived pigments can improve visual appeal while contributing to antioxidant activity. However, natural pigments are often sensitive to pH, oxygen, light, temperature, and processing conditions. Pectin, dietary fibers, and polysaccharides from citrus, apples, mangoes, bananas, and other by-products can support gel formation, stabilization, viscosity, water retention, and texture modification. These functions are technologically valuable; however, they may also alter the mouthfeel, color, aroma, viscosity, or appearance in ways that influence consumer acceptance [60,65,75,76,77,79,80,81,82,85,86,87,88].

For adolescents, these preservative functions can be translated into a simple but scientifically accurate message: food residues may contain compounds that protect food, but protection is not guaranteed. Antioxidant, antimicrobial, coloring, or stabilizing functions must be demonstrated in a specific product, at a specific concentration, and under specific storage conditions. This supports critical food literacy by helping adolescents distinguish between evidence-based preservation and vague claims such as “natural,” “upcycled,” or “clean-label.”

### 4.2. Extraction, Stabilization, and Delivery Technologies

The transition from a by-product to a preservative ingredient requires technological mediation. Bioactive compounds must first be recovered from heterogeneous raw materials, which may vary by species, cultivar, season, processing history, and storage conditions. Conventional extraction methods remain widely used; however, greener and more efficient technologies, including ultrasound-assisted extraction, microwave-assisted extraction, enzyme-assisted extraction, pulsed electric field processing, and supercritical fluid extraction, have gained attention. These methods can improve extraction yield, reduce processing time and solvent use, and better preserve heat-sensitive compounds, although they may also require specialized equipment, process optimization, and economic validation [15,16,17,18,19,20,26,27,57].

Stabilization is equally important because many by-product-derived compounds are sensitive to degradation during processing and storage. Drying, milling, encapsulation, spray drying, freeze-drying, ionic gelation, double emulsions, nanoencapsulation, and edible coatings have been explored to improve stability, protect bioactive compounds, mask undesirable flavors, and control their release. For example, encapsulation approaches have been used for pomegranate peel polyphenols, while citrus-derived pectin films and lemon essential oil systems illustrate the role of packaging-oriented delivery strategies [21,22,65,66,89,101].

Active packaging and edible coatings represent particularly important delivery routes because they allow preservative action to occur at the interface between the food, packaging, and storage environment. Active compounds can be incorporated, coated, immobilized, or released from packaging materials to scavenge oxygen, inhibit microbial growth, reduce oxidation, and maintain quality. This route is especially relevant for clean-label preservation because it may reduce the need for the direct addition of preservatives to the food matrix. However, active packaging raises specific questions related to release kinetics, migration, mechanical and barrier properties, food-contact safety, biodegradability, consumer perception, and environmental performance [12,23,24,54,65,66,101,102].

The technological dimension is especially important for adolescent communication because it prevents simplistic interpretations of the data. By-products do not move directly from “waste” to “food.” They undergo selection, cleaning, extraction, stabilization, formulation, safety assessment, and product testing. This sequence helps adolescents understand that circular innovation depends on science and not on improvisation.

### 4.3. Evidence in Food Matrices

By-product-derived compounds have been tested in several food matrices, including meat and fish products, dairy products, bakery products, beverages, fresh produce, and active-packaging systems. Meat and fish products are among the most relevant matrices because they are susceptible to lipid oxidation, protein oxidation, discoloration, and microbial spoilage. Pomegranate by-products, grape pomace, olive-derived extracts, onion skin powder, lemon by-product extracts, and related compounds have been investigated for their ability to delay oxidation, control microbial growth, and preserve quality during storage. These applications demonstrate their preservative potential; however, sensory attributes, color changes, concentration, and matrix interactions can constrain their practical use [11,12,13,31,68,71,94,96].

Dairy products provide another important application. Citrus pomace, apple pomace, black carrot pomace, beetroot peel extract, and other fruit and vegetable residues have been incorporated into yogurt, Greek yogurt, whey beverages, and cheese products. These applications can increase the fiber, phenolic content, antioxidant activity, pigments, and functional value. However, dairy systems are sensitive to changes in acidity, viscosity, syneresis, color, texture, fermentation behavior and sensory acceptance. The evidence from apple pomace syrup in Greek yogurt, where higher bioactive enrichment did not necessarily correspond to the most acceptable formulation, illustrates a key principle of this review: more by-products are not always better [32,39,40,42,74,87,88].

Bakery products and beverages are attractive matrices because they can incorporate powders, fiber-rich fractions, pomace, and extracts. Apple pomace bread, grape pomace biscuits, pomegranate peel muffins, brewer’s spent grain bakery products, cauliflower by-product beverages, and onion peel-enriched pasta illustrate how by-products can improve the antioxidant capacity, fiber content, phenolic content, color, or functional value. At the same time, they may affect dough rheology, product volume, texture, bitterness, aroma, color, and consumer acceptance of the final product. Therefore, these matrices show both the opportunities and limitations of clean-label valorization [33,34,70,90,98,99].

Fresh produce and active packaging applications further expand preservation perspectives. Edible coatings enriched with natural antioxidant extracts and essential oils have been tested to extend the shelf life of strawberries, while active packaging containing lemon by-product extract or citrus-derived components has been developed to delay lipid oxidation and provide antioxidant and antimicrobial functions. These examples show that preservation can be achieved through both product formulation and packaging design [12,23,24,65,66,101,102].

Across matrices, the evidence points to the same conclusion: food application is the critical test for by-product-derived preservatives. Bioactive composition and in vitro activity are only the beginning of this research. The decisive questions are whether the ingredient or packaging system works in real food, whether it remains stable during storage, whether it improves shelf life or quality, whether it is safe, whether consumers accept it, and whether the sustainability claim is supported by evidence.

### 4.4. Critical Synthesis: From Function to Responsible Application

The pathway from bioactive compounds to food applications is not linear. By-products can provide antioxidant, antimicrobial, coloring, stabilizing, or texturizing compounds; however, their performance depends on extraction, stabilization, formulation, matrix compatibility, safety, sensory acceptance, cost, regulation, and sustainability. This suggests that the scientific value of by-product-derived preservatives should be evaluated using integrated evidence rather than isolated chemical measurements.

This integrated perspective is central to the concept of adolescent food literacy. Adolescents should not simply learn that fruit peels, pomace, or seeds contain “healthy compounds.” They should learn that useful food innovation requires asking how compounds are extracted, whether they remain active, whether they work in real foods, whether they change the taste or texture, whether they are safe, and whether the environmental benefit is demonstrated. In this way, the science of by-product-derived preservation can become a powerful educational tool for developing critical consumers and future citizens capable of engaging responsibly with clean-label and circular economy claims [43,44,49,50,51].

## 5. Critical Translation Challenges: Clean-Label, Safety, Sensory Acceptance, and Sustainability

**Figure 2:**
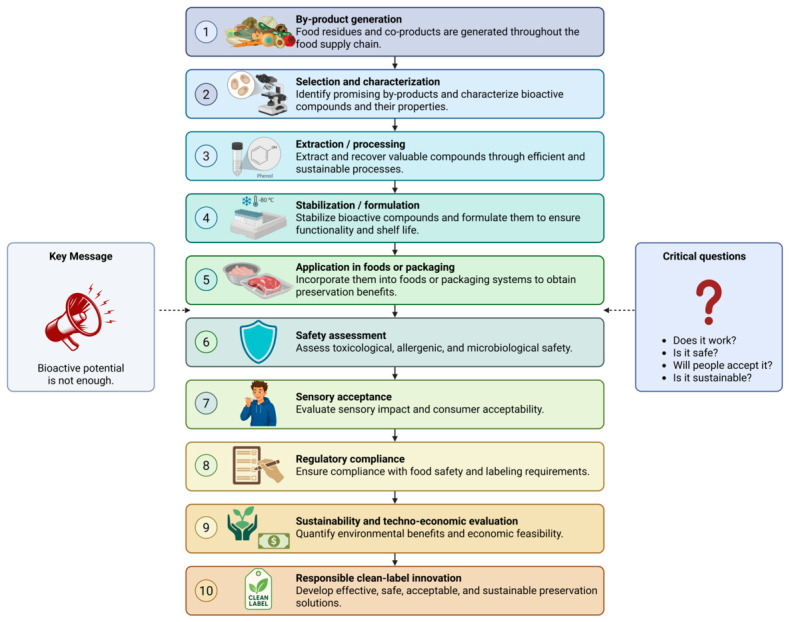
Critical pathway for responsible preservation derived from food by-products. The use of by-product-derived bioactive compounds as natural preservatives requires sequential validation, including selection and characterization, extraction, stabilization, application in foods or packaging, safety assessment, sensory acceptance, regulatory compliance, and sustainability and techno-economic evaluation before responsible clean-label innovation can be achieved. Created in BioRender. Silva, P. (2026) https://BioRender.com/r0gnfkf.

The valorization of food industry by-products as natural preservatives is scientifically promising, but its translation into commercial food products remains complex. The main barriers are not limited to the recovery of bioactive compounds or the demonstration of antioxidant and antimicrobial activities. Successful translation also depends on clean-label expectations, food safety, regulatory classification, standardization, sensory acceptance, consumer trust, economic feasibility and evidence-based sustainability assessment. These challenges are particularly relevant in the context of adolescent food literacy because young consumers are exposed to simplified messages about “natural,” “sustainable,” “upcycled,” and “clean-label” foods (Figure 2). Therefore, a critical educational approach should help adolescents understand that these terms require evidence, not just intention.

### 5.1. Clean-Label Expectations and the Risk of Oversimplification

Consumer demand for foods perceived as natural, minimally processed, and free from synthetic additives has encouraged the food industry to explore natural preservatives and multifunctional ingredients in food products. This trend creates opportunities for by-product-derived compounds, particularly when they can act as antioxidants, antimicrobials, colorants, stabilizers, texturizing agents, or functional ingredients. However, the clean-label concept is not scientifically neutral. Consumer perceptions, familiarity, ingredient names, trust, risk perception, and assumptions about naturalness strongly shape these perceptions [8,9,10].

This creates communication challenges. A by-product-derived ingredient may be technologically useful and environmentally attractive; however, consumers may react negatively if it is associated with “waste” or if its function is unclear. Studies on upcycled foods show that consumers may associate them with sustainability, innovation, and waste reduction but also with concerns about quality, taste, safety, and acceptability [28,29,30]. Therefore, clean-label positioning must avoid the assumption that “natural” or “upcycled” automatically increases consumer acceptance.

For adolescent food literacy, this distinction is important because clean-label claims may refer to different dimensions, including ingredient origin, processing, recognizability, safety, and environmental benefits. Therefore, these claims should be interpreted as prompts for critical questioning, rather than as automatic indicators of product quality.

### 5.2. Safety, Regulation, and Standardization

Safety is one of the most important barriers to the application of by-product-derived preservatives in food. Food industry residues may contain bioactive compounds; however, they may also present risks related to microbial contamination, pesticide residues, heavy metals, mycotoxins, anti-nutritional factors, allergens, or processing contaminants. Their composition can vary according to species, cultivar, season, geographical origin, agricultural practices, processing conditions, storage, and extraction methods. This variability complicates the standardization process and the transition from laboratory-scale extracts to reproducible food ingredients [52,55].

Regulatory classification also poses a challenge. A by-product-derived extract, powder, encapsulated compound, coating, or packaging material may fall under different regulatory categories depending on its composition, intended use, processing method, concentration, and history of consumption. For active packaging, additional questions arise regarding migration, release kinetics, food-contact safety, mechanical properties, barrier properties, and interactions between the active material, food, and storage environment [54,102]. Therefore, real-food validation is essential. Evidence obtained from chemical assays or antimicrobial inhibition tests is insufficient if the ingredient or packaging system is not tested under realistic conditions of formulation, storage and consumption.

Standardization is particularly difficult for residues such as grape pomace, citrus pomace, apple pomace, olive mill wastewater, coffee silverskin, and vegetable peels, because their composition changes with the raw material and processing conditions. Industrial users require predictable ingredients with defined composition, safety specifications, functional performance, and labeling status. Without this, by-product-derived preservatives may remain promising research outputs rather than scalable food solutions.

### 5.3. Sensory Acceptance and Consumer Trust

Sensory quality is a decisive factor in translation processes. Many by-product-derived ingredients are rich in phenolics, fibers, pigments, essential oils, tannins, and bitter compounds. These constituents may improve the antioxidant capacity, color, fiber content, or microbial stability, but they can also modify the flavor, aroma, texture, color, mouthfeel, viscosity, volume, or appearance. This is particularly evident in bakery products, beverages, dairy products, and meat systems, where the same ingredient may improve nutritional or preservative properties while reducing sensory acceptability [32,33,34,70,74,98].

Several examples illustrate this trade-off. Apple pomace syrup increased the total polyphenol, flavonoid, and antioxidant activity in Greek yogurt; however, the most acceptable formulation was not the one with the highest level of apple pomace syrup [74]. Pomegranate peel powder improved the antioxidant and antifungal properties of muffins; however, higher incorporation affected the texture and introduced bitterness [70]. Cauliflower by-product extracts increased the isothiocyanate and phenolic content in apple beverages; however, higher levels negatively affected the taste and aroma [90]. These examples reinforce a central principle: more by-products are not necessarily better.

Consumer trust also depends on transparent communication. If consumers perceive by-product-derived ingredients as unsafe, inferior, or waste-associated, acceptance may decrease. Conversely, acceptance may improve when the ingredient is associated with familiar products, clear benefits, sustainability, safety, and credible labeling [28,29,30]. For adolescents, this creates an important opportunity for critical food literacy: they can learn to distinguish between a product that is genuinely improved by a by-product-derived ingredient and one that uses circularity mainly as a marketing claim.

### 5.4. Economic Feasibility and Industrial Scalability

Economic feasibility is another significant barrier. The recovery of bioactive compounds from by-products often requires collection, transport, sorting, stabilization, drying, milling, extraction, purification, encapsulation, packaging, quality control, and safety testing. Advanced extraction technologies, including supercritical fluid extraction, pulsed electric fields, enzyme-assisted extraction, and microwave-assisted extraction, can improve recovery efficiency but may require high capital investment, specialized equipment, trained personnel, and integration into existing industrial processes [18,26,27].

By-product valorization also depends on a reliable supply chain. Seasonal availability, geographic dispersion, raw material variability, storage stability, and competition with other uses, such as animal feed, composting, energy production, or biorefinery applications, can affect the cost and feasibility of using these by-products. Even when a by-product-derived ingredient performs well in laboratory or pilot-scale studies, industrial adoption requires a predictable supply, scalable processing, regulatory approval, consistent quality, and cost competitiveness with conventional ingredients.

Techno-economic studies have shown that the value of by-product valorization depends strongly on process design, product yield, energy demand, solvent recovery, equipment costs, and the integration of residual biomass streams [36,37,38]. Therefore, the commercial translation of natural preservatives from by-products cannot rely solely on biological activity; it must also demonstrate economic viability and operational compatibility with food industry systems.

### 5.5. Sustainability Claims: Evidence or Narrative?

The sustainability argument is central to by-product valorization; however, it must be demonstrated rather than assumed. Using by-products can reduce waste disposal burdens, create value-added ingredients, and support circular food systems. However, environmental benefits depend on the entire process, including collection, transport, drying, extraction, solvent use, water consumption, energy demand, stabilization, packaging, storage, and the fate of the remaining residues. A by-product-derived preservative is not automatically sustainable simply because its raw material originates as waste.

Therefore, life cycle assessments, techno-economic analyses, and eco-efficiency studies are essential to distinguish evidence-based circularity from sustainability narratives. Recent studies have shown that environmental and economic outcomes depend on process design, scale, product yield, extraction technology, and the integration of co-products and residual biomass streams [35,36,37,38]. This is especially important for technologies that require solvents, high-energy inputs, specialized equipment, or additional packaging materials.

The same critical approach applies to active packaging and biodegradable film. A packaging system may extend the shelf life and reduce food waste, but it may also introduce new materials, processing requirements, migration concerns, or end-of-life challenges. Therefore, sustainability should be assessed using measurable indicators, not only through terms such as “green,” “natural,” “bio-based,” or “circular” [54,102].

### 5.6. Implications for Adolescent Food Literacy

The translation challenges described above also present educational opportunities. They provide adolescents with concrete criteria for evaluating food innovation: whether an ingredient performs a clear function, whether it was tested in a real food product, whether it is safe and acceptable, whether the label is transparent, and whether the sustainability claims are supported by evidence. This approach links technological validation with critical food literacy and prepares the transition to Section 6, where these implications are developed from an educational perspective [43,44,49,50,51].

## 6. Adolescents as Agents of Change: Translating Preservation Science into Food Literacy

**Figure 3:**
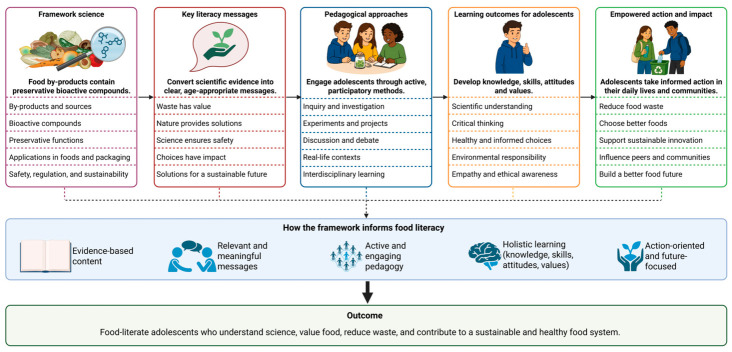
Integration of the conceptual framework into adolescent food literacy. Scientific evidence on food by-products and preservative bioactive compounds can be translated into age-appropriate literacy messages, active pedagogical approaches, learning outcomes, and empowered actions that support critical food choices and more sustainable food systems. Created in BioRender. Silva, P. (2026) https://BioRender.com/ulxnt8n.

The scientific evidence on food industry by-products, natural preservatives, clean-label innovation, and circular food systems has educational value that extends beyond the field of food technology. For adolescents, this topic can become a concrete way to understand how food systems generate residues, how science can transform selected by-products into useful ingredients, and why claims such as “natural,” “clean-label,” “upcycled,” or “sustainable” must be interpreted critically. In this review, adolescents are not approached as passive recipients of dietary advice but as current and future consumers, household influencers, school community members, peer communicators, digital citizens, and emerging participants in food system transformation. This translational pathway is summarized in Figure 3.

### 6.1. Why Adolescents Matter in Circular Food Systems

Adolescence is a particularly important period for developing food literacy. During this life stage, young people progressively gain autonomy over food choices, are more exposed to food marketing and digital communication, and begin to develop attitudes and habits that may influence future dietary behaviors. Therefore, food literacy in adolescence is not limited to knowing which foods are healthy. It also includes the ability to understand food systems, interpret food information, evaluate claims, make decisions in social contexts, and connect personal choices to broader environmental and societal consequences [43,49].

Adolescents should not be considered a homogeneous group. Their food knowledge, autonomy, interests, media exposure, and ability to interpret food claims may vary according to age, sex, cultural background, socioeconomic context, family food practices, school curriculum, and national food environments. Therefore, the educational implications proposed in this review should be adapted to the developmental stage, prior knowledge, cultural context, and learning environment of different adolescent groups. For younger adolescents, the focus may be on familiar examples and basic label interpretation; for older adolescents, more complex discussions can include food processing, active packaging, consumer acceptance, regulation, and sustainability evidence.

The relevance of adolescents is also relational in nature. They are influenced by families, peers, school food environments, social media, and consumer trends. School-based food systems education has shown that adolescents can engage with topics such as diet quality, sustainability, and food waste when these issues are made concrete and connected to their everyday contexts [44]. Moreover, adolescents’ perceptions of sustainable diets often include myths, uncertainties, and partial understandings, which reinforces the need for educational strategies that combine scientific accuracy with accessible communication [48].

By-product-derived preservation offers a particularly useful educational theme because it connects familiar foods to the hidden parts of the food system. Orange juice generates citrus peels and pomace; wine and grape juice generate grape pomace; apple juice generates apple pomace; yogurt and bakery products can incorporate fruit or vegetable residues; and packaging can include active compounds derived from by-products. These examples help adolescents understand that food waste is not only what remains on the plate. It also includes the processing residues generated before the food reaches the consumer. This broader understanding can support a more systemic and responsible perspective on food waste.

### 6.2. What By-Product Valorization Can Teach Adolescents

The first educational message is that some food industry by-products may contain valuable compounds. Peels, pomaces, seeds, skins, and other residues may contain phenolics, flavonoids, carotenoids, anthocyanins, essential oils, pectin, dietary fibers, and other compounds with antioxidant, antimicrobial, coloring, stabilizing, and texturizing functions. However, this message must be communicated with caution. The presence of bioactive compounds does not imply that a residue is automatically edible, safe, effective, or sustainable. Selected by-products can become useful ingredients only after characterization, processing, stabilization, safety assessment, formulation, and testing in actual food systems.

From an educational perspective, this review proposes that messages for adolescents should avoid suggesting that food waste can simply be eaten or that natural preservatives are inherently safer. Instead, the reviewed evidence supports a more nuanced message: selected residues may become safe and functional ingredients only when their transformation is supported by characterization, processing, testing, and regulation. This approach avoids simplistic narratives and encourages critical thinking. It also helps adolescents distinguish between food waste prevention, by-product valorization, upcycling, and unsafe or inappropriate reuses.

The second educational message is that preservation is a part of sustainability. Shelf-life extension can reduce food waste by slowing oxidation, microbial spoilage, discoloration, texture degradation, and quality loss. However, preservation requires resources. Extraction, drying, encapsulation, packaging, storage, and transport may use energy, water, solvents, materials, and specialized equipment to complete the process. Therefore, a circular food solution should be evaluated not only by the origin of the ingredient but also by evidence that it reduces waste, maintains safety and quality, is acceptable to consumers, and has a defensible environmental balance [35,36].

The third educational message concerns labels and claims. Adolescents are exposed to food labels, front-of-pack information, environmental claims, clean-label claims, and marketing narratives that promote food products. Research on young consumers shows that labels and sustainability claims can influence perceptions and choices, but this influence depends on how information is framed, understood, and trusted [50]. By-product-derived preservatives create an opportunity to teach adolescents to ask what a claim means. Does “natural” refer to origin, processing, safety, or consumer perception? Does “upcycled” mean that waste was reduced, or only that a by-product was included? Does “clean-label” mean fewer additives, recognizable ingredients, or better health and sustainability? These questions help transform label reading into a critical food literacy.

### 6.3. From Scientific Evidence to Critical Questions

Accordingly, Table 3 should not be interpreted as direct evidence that these questions improve adolescent behavior but as an educational translation of the reviewed evidence into critical questions that can guide future food literacy interventions. The purpose is not to expect adolescents to master food technology, extraction methods, regulatory frameworks, or life-cycle assessment in technical detail. Rather, the aim is to translate these areas into age-appropriate questions: What does this label mean? Was the ingredient tested? Does this affect the taste or safety? Is the sustainability claim supported by evidence? This approach allows complex scientific content to be adapted to different educational levels.

Figure 2 and Figure 3 may also support educational activities by helping readers distinguish three related but different concepts: by-product-derived ingredients, which are recovered from food industry residues; active packaging, which can preserve food through the packaging system rather than direct ingredient addition; and clean-label claims, which refer to how ingredients or technologies are communicated to consumers. Table 4 builds on this distinction by providing illustrative label-reading examples that help translate these concepts into practical questions about origin, function, safety, acceptability, and sustainability evidence.

This question-based approach is useful because it moves adolescents from passive knowledge acquisition to active interpretation of the information. This aligns with the broader purpose of food literacy: developing the ability to make informed, contextualized, and critical food-related decisions [43,49]. In the context of by-product valorization, this means learning to connect chemistry, food technology, sensory quality, food safety, environmental sustainability, and consumer communication.

### 6.4. Communication Principles for Adolescent Food Literacy

Translating this topic for adolescents requires accurate, concrete, and question-oriented communication. First, messages should avoid oversimplification: selected by-products may become useful ingredients only when they are characterized, processed, tested, and appropriately regulated. Second, communication should use familiar examples, such as citrus peels, apple pomace, grape pomace, yogurt, bread, fruit juices, chocolate, coffee, and food packaging, to make invisible food system processes more visible. Third, safety, sensory quality, sustainability, and consumer acceptance should be discussed together because sustainable food innovation must also be safe, acceptable, and evidence-based. Finally, label literacy should be treated as a critical skill: claims such as “natural,” “clean-label,” “upcycled,” “sustainable,” “green,” or “circular” should be starting points for questions rather than final proof of quality [50,51].

### 6.5. Adolescents as Agents of Change

Adolescents can become agents of change in several complementary ways. At the individual level, people can reduce avoidable food waste, improve storage practices, interpret food labels more critically, and make more informed food choices. At the household level, they can influence conversations about food purchasing, leftovers, storage, packaging and waste reduction. At the school level, they can participate in food literacy projects, sustainability campaigns, canteen initiatives, science communication activities, and citizen science approaches to reduce food waste. At the peer and digital levels, they can communicate evidence-based messages and challenge misleading or exaggerated claims to the public [43,44,47,48,49].

The topic of by-product-derived preservation is especially powerful because it links everyday food choices to scientific reasoning. This shows that food sustainability is not only about eating “better” foods but also about understanding how foods are produced, processed, preserved, packaged, labeled, marketed, consumed, and discarded. It also shows that innovation requires a trade-off. A product may reduce waste but have sensory limitations; a natural extract may be promising but unstable; active packaging may extend shelf life but require evaluation of material impacts; an upcycled ingredient may be sustainable in principle but rejected by consumers if communication is unclear [29,30,35,36,54,74].

Positioning adolescents as agents of change means giving them the tools to ask better questions. They do not need to become food technologists, but they can learn to think critically about food systems. They can ask whether a claim is supported by evidence, whether a natural ingredient is safe and effective, whether a by-product has been tested in a real food matrix, whether sensory quality was considered, and whether sustainability was measured rather than being assumed. This is the distinctive educational contribution of the present review: food by-product valorization is not only a technological pathway for clean-label preservation but also a platform for developing critical, evidence-based, and sustainability-oriented food literacy among adolescents [43,44,48,49,50,51].

## 7. Limitations

This review has some limitations. First, it is a narrative review and did not follow a systematic review protocol; therefore, it does not provide an exhaustive or quantitative synthesis of all studies on food industry by-products, natural preservatives, and adolescent food literacy. Second, although this manuscript integrates evidence from food science, consumer research, sustainability assessment, and food literacy, the educational framework proposed here has not been empirically tested. Therefore, the review does not demonstrate that adolescents’ knowledge, purchasing decisions, or food behaviors change as a result of exposure to this topic. Third, adolescents are discussed as a broad population group, although food literacy, food choices, and responses to sustainability or clean-label claims may differ by age, sex, socioeconomic context, culture, country, school curriculum, family practices, and digital media exposure. Fourth, this review focuses mainly on the conceptual and educational potential of by-product-derived preservation and does not provide a market-level assessment of the economic feasibility of specific ingredients or products. These limitations highlight the need for future empirical studies to evaluate both technological translation and educational implementation in defined adolescent populations.

## 8. Future Perspectives

Future research should focus on translating promising laboratory findings on by-product-derived natural preservatives into validated food applications. The first priority is real-food validation. Bioactive extracts, powders, edible coatings, and active packaging systems should be tested in specific food matrices under realistic processing, storage, distribution, and consumption conditions. This includes assessing the dose, stability, release behavior, preservative efficacy, sensory impact, food safety, and shelf-life extension across different product categories.

The second priority is industrial scalability and techno-economic feasibility. Many by-product-derived ingredients require collection, sorting, stabilization, extraction, purification, formulation, packaging, and quality control. Therefore, future studies should evaluate process standardization, raw material variability, supply chain logistics, cost competitiveness, energy and water use, solvent recovery, regulatory classification, and integration into existing food industry systems. Life cycle assessment and eco-efficiency analyses should be incorporated to determine whether by-product valorization produces measurable environmental benefits, rather than only supporting circularity narratives.

The third priority concerns consumer acceptance and communication. More research is needed to understand how consumers, including adolescents and families, interpret terms such as “natural,” “clean-label,” “upcycled,” “circular,” and “sustainable.” Studies should examine trust, perceived safety, sensory expectations, willingness to consume, willingness to pay, and the role of transparent labeling in this context. This is particularly important because consumer acceptance may determine whether technically promising ingredients become viable food solutions for the food industry.

Finally, future studies should explore the implementation of this topic in adolescent food literacy education. School-based interventions, participatory activities, label interpretation exercises, food waste projects, citizen science approaches, and digital communication strategies can be used to evaluate whether adolescents improve their understanding of food systems, preservation, safety, sustainability claims, and critical food choices. These educational studies should move beyond knowledge acquisition and assess critical thinking, decision-making, communication skills, and potential changes in adolescents’ household, school, peer, and digital food practices.

In practical terms, the framework proposed in this review can support the future development of classroom activities, label interpretation exercises, food waste projects, discussions on natural and clean-label claims, and science communication resources for adolescents. These activities should not aim to promote specific by-product-derived ingredients but to help adolescents understand how food innovations are evaluated and communicated. Their effectiveness should be tested in future educational interventions that assess not only knowledge acquisition but also critical thinking, label interpretation, trust in claims, communication skills, and food-related decision-making.

## 9. Conclusions

Food industry by-products are promising sources of natural preservative compounds for clean-label food applications, shelf-life extension, and circular food systems. Fruit and vegetable pomaces, peels, seeds, skins, cereal residues, olive oil and wine by-products, coffee silverskin, cocoa residues, and other agro-industrial side streams may contain phenolic compounds, flavonoids, carotenoids, anthocyanins, essential oils, pectin, dietary fibers, polysaccharides, and other compounds with antioxidant, antimicrobial, coloring, stabilizing, and texturizing properties. These materials can support the development of multifunctional ingredients, edible coatings, active packaging systems, and food formulations that reduce reliance on synthetic additives and contribute to waste valorization [52,54,55].

However, the use of by-products as natural preservatives should not be considered a simple or automatically sustainable solution. The presence of bioactive compounds does not guarantee preservative efficacy, food safety, sensory acceptance, regulatory suitability and environmental benefits. Translating these findings into food applications requires standardized characterization of raw materials, appropriate extraction and stabilization technologies, validation in real food matrices, safety assessment, sensory optimization, consumer acceptance studies, economic feasibility analysis, and evidence-based sustainability evaluation. In this context, clean-label and circular economy claims should be treated critically and supported by measurable evidence rather than being used only as marketing language [25,26,27,28,29,30,35,36,37,38].

The distinctive contribution of this review is the integration of adolescent food literacy into the discussion of by-product-derived preservation. Adolescents are not only future consumers; they are also current food decision-makers, family influencers, peer communicators, digital citizens, and emerging participants in food system transformation. Translating the science of food by-product valorization for adolescents can help them understand that food waste is not only something to be reduced but also, in selected cases, a potential resource when managed safely and scientifically. Simultaneously, it can help them recognize that natural, upcycled, clean-label, or circular products are not automatically safe, effective, acceptable, or sustainable [43,44,48,49,50,51].

By connecting food preservation, clean-label innovation, sensory quality, food safety, labeling, sustainability, and circular economy principles, this topic offers a powerful platform for enhancing food literacy. Adolescents can be encouraged to ask which by-product was used, which compound is responsible for the preservative effect, how it was processed, whether it was tested in a real food system, whether safety and sensory acceptance were evaluated, and whether sustainability claims were supported by evidence. In this sense, food industry by-products are not only technological resources for preservation, but also educational tools that can empower adolescents to become more critical consumers and agents of change in more sustainable food systems [43,44,49].

## Figures and Tables

**Table 1:** Conceptual framework linking food by-product valorization, clean-label preservation, circularity, and adolescent agency.

Framework Dimension	Scientific and Technological Focus	Critical Issue for Review Analysis	Implication for Adolescent Food Literacy and Agency	Key Supporting References
By-product generation	Food processing generates residues such as peels, seeds, skins, pomace, stems, leaves, and other fractions.	These materials are often treated as waste or used in low-value applications despite their possible functional value.	Food waste is not only a disposal problem; selected residues can become resources when safely and scientifically managed.	[52,55]
Bioactive potential	By-products may contain phenolic compounds, flavonoids, carotenoids, anthocyanins, dietary fibers, vitamins, minerals, essential oils, and other phytochemicals.	Bioactive composition varies according to species, cultivar, seasonality, processing, storage, and extraction conditions.	Not all residues are equivalent; scientific testing is needed to identify useful compounds and distinguish evidence from assumptions.	[52,55]
Preservative function	Bioactive compounds may delay oxidation, inhibit microbial growth, reduce browning, stabilize color, or improve functional properties.	Activity observed in extracts or model systems may not translate directly into real foods.	A compound is only useful as a preservative if it works safely and effectively in a real food product.	[52,54,55]
Extraction and processing	Selection, cleaning, drying, extraction, purification, concentration, and formulation are often required.	Green technologies may improve recovery but raise questions about cost, scalability, standardization, regulatory compliance, and industrial feasibility.	Science and technology are needed to transform residues into safe and useful ingredients; innovation must be evaluated critically.	[55]
Stabilization and delivery	Encapsulation, spray drying, freeze-drying, coacervation, ionic gelation, nanoencapsulation, edible coatings, and active packaging can protect and deliver bioactives.	Natural compounds may be unstable under light, oxygen, pH, temperature, and processing conditions.	“Natural” does not automatically mean stable, safe, or effective; formulation and testing matter.	[54,55]
Food application	By-product-derived preservatives have been tested in meat, dairy, bakery products, beverages, fresh produce, and packaging systems.	Each food matrix has specific technological, safety, sensory, regulatory, and consumer acceptance requirements.	Food innovation must consider taste, texture, safety, labeling, affordability, and acceptance, not only sustainability.	[42,52,55]
Circularity and sustainability	Valorization can reduce disposal burdens, create value-added ingredients, and support shelf-life extension.	Sustainability claims must be supported by evidence, including energy use, solvents, water, packaging, transport, scale-up, and actual waste reduction.	Circular economy is not only a slogan; adolescents should learn to question whether sustainability claims are evidence-based.	[42,52,55]
Industrial and market translation	Promising laboratory or pilot-scale results must be scaled, regulated, labeled, priced, and accepted by consumers.	Translation is limited by variability, safety concerns, cost, regulation, sensory performance, consumer trust, and market positioning.	Adolescents can become critical consumers who distinguish scientific innovation from marketing claims.	[54,55]
Adolescent agency	Adolescents influence household choices, school food environments, peer norms, digital communication, and future consumer expectations.	Educational communication must avoid simplistic messages such as “all waste can be eaten” or “natural is always safer.”	Adolescents can reduce waste, question labels, communicate evidence-based messages, and support safe, sustainable innovation.	[43,44,49]

**Table 2:** Main food industry by-products investigated as sources of natural preservatives or functional compounds and their relevance for adolescent food literacy.

By-Product Group	Examples	Main Compounds/Functions	Food Applications Reported	Critical Issue	Food Literacy Message for Adolescents
Citrus by-products	Orange, lemon, grapefruit, mandarin and lime peels, pulp, seeds, pomace	Flavonoids, polyphenols, carotenoids, vitamin C, pectin, dietary fiber, essential oils, limonene	Edible films, active packaging, bakery products, dairy drinks, yogurt, beverages, coatings	Bitterness, aroma intensity, essential oil volatility, dose, sensory impact, regulatory use	Citrus waste can contain useful compounds, but these must be extracted, stabilized, and tested
Grape by-products	Grape pomace, skins, seeds, stems	Anthocyanins, flavanols, proanthocyanidins, phenolic acids, fiber, seed oil	Meat products, bakery products, functional ingredients, antioxidant extracts	High variability by cultivar and processing; color and astringency effects	A familiar food chain can generate hidden residues with functional value
Pomegranate by-products	Peel, pomace, rind	Ellagitannins, punicalagin, ellagic acid, tannins, phenolics, flavonoids	Muffins, meat, fish products, films, extracts	Bitterness, color, concentration, sensory acceptance	Strong bioactivity must be balanced with taste and safety
Apple by-products	Apple pomace, peel, pulp residues, seeds	Pectin, dietary fiber, chlorogenic acid, quercetin, catechin, procyanidins	Yogurt, Greek yogurt, bakery products, fiber-rich foods	Moisture, microbial spoilage, texture, optimal concentration	More sustainable ingredients still need consumer acceptance
Mango and banana by-products	Mango peel, mango seed kernel, banana peel	Fiber, pectin, carotenoids, vitamin C, vitamin E, sterols, squalene, phenolics	Dairy products, stabilizers, fat replacers, pectin, functional ingredients	Processing, stability, concentration, sensory effects	A peel may be a resource, but not all peels are ready-to-eat ingredients
Vegetable by-products	Tomato peels/seeds, carrot pomace, beetroot peel, broccoli leaves, cauliflower residues, potato peel, onion peel	Lycopene, β-carotene, betalains, glucosinolates, isothiocyanates, phenolics, fiber	Beverages, yogurt, whey products, natural colorants, antioxidant ingredients	Pigment instability, off-flavors, contaminants, matrix interactions	Natural color and preservation claims should be questioned scientifically
Olive by-products	Olive pomace, olive leaves, olive paste, olive mill wastewater	Hydroxytyrosol, tyrosol, oleuropein derivatives, secoiridoids, phenolics	Meat, oils, bakery, dairy-related applications	Bitterness, astringency, regulatory and sensory issues	Traditional food systems also generate residues that can be valorized
Cereal, brewing, coffee and cocoa residues	Cereal bran, brewer’s spent grain, coffee silverskin, cocoa husk/shell	Fiber, β-glucans, phenolic acids, chlorogenic acids, methylxanthines, antioxidants	Bakery products, functional ingredients, beverages, fortification	Texture, flavor, caffeine/methylxanthine content, consumer perception	Popular foods create invisible residue streams that can be discussed critically

**Table 3:** Translating scientific evidence on by-product-derived preservatives into adolescent food literacy questions.

Scientific Concept	Food Literacy Message for Adolescents	Critical Question Adolescents Can Ask
Food processing generates by-products	Food waste is not only what remains on the plate; residues are also generated during processing.	Where does this residue come from in the food chain?
By-products may contain bioactive compounds	Some residues contain compounds that may help protect food from oxidation, spoilage, or quality loss.	Which compound is responsible for the effect?
Natural compounds require testing	Natural origin does not automatically mean safe, stable, or effective.	Was this ingredient tested for safety and efficacy?
Preservative action is matrix-dependent	A compound that works in a laboratory test may not work in yogurt, bread, meat, beverages, or packaging.	Was it tested in a real food product?
Dose matters	Higher amounts may increase bioactivity but worsen taste, texture, color, or acceptance.	What amount was used, and did it affect sensory quality?
Processing matters	Extraction, drying, encapsulation, and packaging influence stability and performance.	How was the by-product processed before use?
Active packaging can preserve food	Preservation can occur through packaging as well as through ingredients added to food.	Is the packaging active, and how does it protect the food?
Clean-label claims need interpretation	A clean-label does not automatically mean safer, healthier, or more sustainable.	What does the clean-label claim actually mean?
Circularity requires evidence	Using a by-product does not automatically prove sustainability.	Is there evidence of waste reduction or lower environmental impact?
Consumers matter	A product must be safe, effective, acceptable, affordable, and understandable.	Would people trust, buy, and consume this product?

**Table 4:** Illustrative label-reading examples linking by-product ingredients, active packaging, and clean-label claims.

Label Element Adolescents May Encounter	Possible By-Product Source	Critical Interpretation Question
Citrus fiber, citrus pectin, or lemon extract	Citrus peels, pomace, or processing residues	Is this ingredient used for texture, stabilization, antioxidant activity, flavor, or preservation?
Apple fiber or apple pomace ingredient	Apple juice or cider processing residues	Does the label explain the ingredient’s function, or is it mainly presented as a sustainability claim?
Grape skin extract or grape pomace powder	Wine or grape juice by-products	Was it added for antioxidant activity, color, fiber enrichment, or another technological function?
Cocoa shell or coffee silverskin fiber	Cocoa or coffee processing residues	Is the ingredient nutritionally or technologically useful, and is its origin clearly communicated?
Active packaging with plant extract or essential oil	Fruit, vegetable, or agro-industrial by-products	Does packaging protect the food, and is there evidence that it extends shelf life safely?
“Natural,” “clean-label,” “upcycled,” or “sustainable” claim	May refer to ingredient origin, processing, packaging, or environmental positioning	What evidence supports the claim, and does it relate to safety, efficacy, sensory quality, or sustainability?

Note: These examples are illustrative and are not intended to refer to specific commercial products. They are based on studies addressing by-product valorization, active packaging, consumer acceptance of upcycled foods, and clean-label communication [28,29,30,52,54,55,56,60,65,66,97,100,102].

## Data Availability

The original contributions presented in this study are included in the article. Further inquiries can be directed to the corresponding author.
